# Structural basis for ligand recognition by a Cache chemosensory domain that mediates carboxylate sensing in *Pseudomonas syringae*

**DOI:** 10.1038/srep35198

**Published:** 2016-10-13

**Authors:** Jodi L. Brewster, James L. O. McKellar, Thomas J. Finn, Janet Newman, Thomas S. Peat, Monica L. Gerth

**Affiliations:** 1 Department of Biochemistry, University of Otago, Dunedin, 9054, New Zealand; 2 Biomedical Manufacturing Program, Commonwealth Scientific and Industrial Research Organisation (CSIRO), Parkville, Victoria, 3052, Australia

## Abstract

Chemoreceptors enable bacteria to detect chemical signals in the environment and navigate towards niches that are favourable for survival. The sensor domains of chemoreceptors function as the input modules for chemotaxis systems, and provide sensory specificity by binding specific ligands. Cache-like domains are the most common extracellular sensor module in prokaryotes, however only a handful have been functionally or structurally characterised. Here, we have characterised a chemoreceptor Cache-like sensor domain (PscD-SD) from the plant pathogen *Pseudomonas syringae* pv. *actinidiae* (*Psa*). High-throughput fluorescence thermal shift assays, combined with isothermal thermal titration calorimetry, revealed that PscD-SD binds specifically to C_2_ (glycolate and acetate) and C_3_ (propionate and pyruvate) carboxylates. We solved the structure of PscD-SD in complex with propionate using X-ray crystallography. The structure reveals the key residues that comprise the ligand binding pocket and dictate the specificity of this sensor domain for C_2_ and C_3_ carboxylates. We also demonstrate that all four carboxylate ligands are chemoattractants for *Psa*, but only two of these (acetate and pyruvate) are utilisable carbon sources. This result suggests that in addition to guiding the bacteria towards nutrients, another possible role for carboxylate sensing is in locating potential sites of entry into the host plant.

Motile bacteria are attracted by certain chemicals and repelled by others, a behaviour that enables them to navigate towards favourable niches for growth and survival^1^,^2^. This process, chemotaxis, is mediated by arrays of transmembrane chemoreceptors (also known as methyl-accepting chemotaxis proteins (MCPs)). These arrays can include several different kinds of chemoreceptors, each able to recognise either one or many specific attractants and repellents. Chemoreceptor proteins bind ligands (either directly or indirectly) and transmit a signal to downstream chemotaxis machinery in order to control flagellar rotation and bacterial motility^3^,^4^. Chemoreceptors typically have three structurally and functionally discrete domains: a periplasmic sensor domain, a transmembrane HAMP domain, and a cytoplasmic signalling domain. For chemoreceptors with this domain architecture, the specificity of a chemotactic response is determined by the periplasmic sensor domain.

The signalling domains of chemoreceptors are highly conserved between different species and in fact, are often used to identify putative chemoreceptor genes from genomic sequences. In contrast, the sensor domains are highly diverse and vary significantly from species to species and from receptor to receptor^5^,^6^. A veritable alphabet soup of sensor domains have been identified in chemoreceptors: including GAF, PAS, NIT, Chase, Cache and four-helix bundle domains. Arguably the best studied are the four-helix bundle domains found in the chemoreceptors of enteric bacteria such as *Escherichia coli* and *Salmonella* species. However, a recent bioinformatics study has shown that the most common extracellular sensor domains found in prokaryotes are actually Cache (Calcium channels and chemotaxis receptors) domains^7^. Cache domains are homologous to (though distinct from) the common intracellular Per/Arnt/Sim (PAS) sensor domains^7^. PAS domains are known to bind an incredibly diverse range of ligands while maintaining the conserved PAS fold^8^. At this time, only a handful of Cache domains have been characterised, and most of these are highly specific for monocarboxylates. Currently, it is unclear if Cache domains are more specialised than PAS, or rather their functional diversity is merely under-explored.

To address this gap in knowledge, we set out to characterise two Cache domains from the plant pathogen *Pseudomonas syringae* pv. *actinidiae* (*Psa*). *Psa* is a Gram-negative, motile bacterium with polar flagella^9^. *Psa* is also a destructive pathogen of kiwifruit that is currently causing severe economic losses to kiwifruit industry worldwide^9^. It invades host plant tissues through natural openings (*e.g*. stomata, lenticels), or via lesions or wounds^10^. Colonisation of the host plant results in leaf spotting, canker formation, dieback and in severe cases, plant death^10^. For many plant pathogens, chemotaxis allows the bacterium to navigate over the plant surface in order to locate potential entry sites^11^,^12^,^13^. Plant-associated microbes are often attracted to a wide range of molecules found in host exudates, such as amino acids, organic acids and sugars^11^,^14^.

Fundamentally, it is the repertoire of chemoreceptor proteins in a microorganism that determines the environmental signals to which it responds. Bacteria faced with complex environments tend to have diverse chemosensory systems with numerous chemoreceptors, in order to sense the abundance of chemical stimuli^2^,^6^. It comes as no surprise then that *Psa* has a large number of putative chemoreceptors encoded in its genome (43, as compared to four in *E. coli*)^15^, and that these receptors are predicted to possess a wide variety of sensor domain folds^16^. Of these, six are predicted to have Cache-like sensor domains. We have recently characterised three of these chemoreceptors; each has a double-Cache sensor domain, and each binds a specific subset of amino acids^16^.

Here, we report the structural and functional characterisation of a Cache chemoreceptor sensor domain from *Psa*. Our results demonstrate that this chemoreceptor, designated PscD, has a single Cache sensor domain that recognises specific C_2_ and C_3_ carboxylate ligands. Of the ligands probed, PscD has the highest affinity for glycolate, and this ligand is also the strongest chemoattractant for *Psa*. As far as we know, PscD represents the first chemoreceptor demonstrated to recognise glycolate - an important plant metabolite that is also involved in the control of stomatal opening in leaves^17^. We have solved the structure of the PscD sensor domain using X-ray crystallography, and compared it with other recently determined Cache domain structures. Together, these data advance our understanding of the specificity of ligand recognition in chemoreceptor sensor domains.

## Results

### Two *Psa* chemoreceptors have single Cache sensor-domains: PscD and PscE

Using the newly defined Cache domain hidden Markov model^7^,^18^, we identified two chemoreceptors in the *Psa* NZ-V13 genome predicted to have single Cache-type sensor domains (sCache_2 family): PscD and PscE (corresponding to loci Psa_018780 and Psa_011445, respectively). The chemoreceptor sequences were analysed using TOPCONS^19^ in order to identify the transmembrane helices and therefore which residues comprise the sensor domain(s). The sensor domains of PscD and PscE were predicted to consist of residues Gln31–Trp188 and Met31–Tyr206, respectively. A comparison of their sequences shows they exhibit a pairwise sequence identity of only 23% (39% similarity). Given that just a small number (~1–3) of amino acid residue differences in the ligand-binding site can dramatically alter the ligand specificity of chemoreceptor sensor domains^16^,^20^,^21^, this high degree of sequence variation suggests that PscD-SD and PscE-SD have different ligand binding repertoires.

In order to investigate their function, the two predicted sensor domains were cloned and expressed in *E. coli*. PscD-SD was expressed to very high levels and was soluble; we routinely obtained >20 mg of purified PscD-SD per liter of bacterial culture. In contrast, PscE-SD was completely insoluble under all conditions tested. All subsequent experiments were therefore conducted with PscD-SD.

### The PscD sensor domain binds to C_2_ and C_3_ carboxylates

We used high-throughput fluorescence thermal shift (FTS) assays (as described previously^16^) to screen for potential ligands of PscD-SD. FTS assays are based on the principle that ligands stabilise proteins upon binding, causing an increase in the melting temperature of the protein^22^,^23^. Binding can therefore be rapidly screened by measuring the difference in the temperature midpoint of unfolding (ΔT_m_) of a protein in the presence and absence of potential ligands. In our FTS assays, the T_m_ was measured by monitoring an increase in fluorescence of a dye with affinity for hydrophobic parts of the protein (*i.e*. SYPRO Orange), which are exposed as the protein unfolds. We screened the recombinant PscD-SD protein in FTS assays against the compounds in Biolog Phenotype Microarray (PM) plates 1 and 2 (Supplementary Tables 1 and 2). Each plate contains 95 different compounds (*e.g*., organic acids, sugars) and a water (no ligand) control. For use in FTS assays, the compounds were resuspended directly in water, thus generating a library of biologically relevant potential ligands. Though the exact concentrations of individual compounds in the Biolog PM plates are proprietary and not released by the manufacturer, we have found these plates to be useful for qualitative, high-throughput screening.

**Figure 1:**
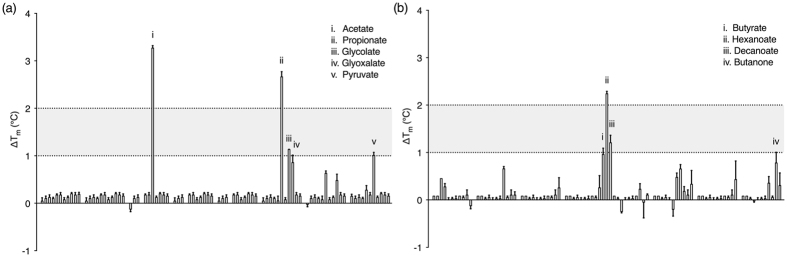
High-throughput fluorescence-based thermal shift assays of PscD-SD with 190 potential ligands from Biolog plates PM1 (**a**) and PM2 (**b**). Each histogram indicates the ΔT_m_, calculated as the T_m_ in the presence of ligand minus the T_m_ in the absence of ligand. The ligands identified as potential hits are labelled. The values graphed are the mean and standard error from three independent experiments.

In the absence of ligands, the PscD-SD protein displayed a T_m_ of 56.1 ± 0.3 °C. Of the 190 potential ligands screened, acetate, propionate and hexanoate resulted in the largest temperature shifts, with ΔT_m_ values of >2 °C (Fig. 1). An additional six potential ligands were identified that produced marginal stabilising effects in the range of ~1–2 °C (Fig. 1). Stock solutions of the nine potential ligands identified from the FTS Biolog screening were prepared at known concentrations, and the hits were re-screened using FTS assays with increasing ligand concentrations (given that T_m_ will increase with fractional ligand occupancy). Increasing concentrations of butyrate, hexanoate, glyoxylate and butanone did not measurably increase the thermal stability of the protein in our assays (Supplementary Figure 1), indicating that they are not ligands for PscD-SD. In contrast, increasing concentrations of glycolate, pyruvate and decanoate all resulted in increased ΔT_m_ values (Supplementary Figure 1). Combined, the high-throughput screens identified five potential ligands for PscD-SD: glycolate, acetate, propionate, pyruvate and decanoate.

**Figure 2:**
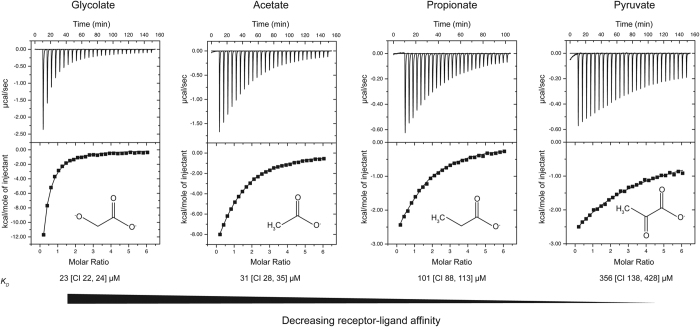
Representative isothermal titration calorimetry of PscD-SD with different ligands. The upper panels show raw titration data, the lower panels are the integrated and dilution corrected peak areas of the titration data. The dissociation constants (*K*_D_) shown are the global fit of at least two independent experiments, and the values in brackets are the 95% confidence intervals.

Next, we used isothermal titration calorimetry to quantitatively assess the binding of these putative ligands to the sensor domain of PscD. The titration curves of purified PscD-SD show direct binding with C_2_ (glycolate and acetate) and C_3_ (propionate and pyruvate) carboxylates with micromolar affinity (Fig. 2). The C_2_ ligands glycolate and acetate bind directly to PscD-SD with *K*_D_ values of of 23 μM [95% confidence limits (CI) 22, 24 μM] and 31 μM [CI 28, 35 μM], respectively. A *K*_D_ of 101 μM [CI 88, 113 μM] was determined for propionate. The affinity of PscD-SD for pyruvate is comparatively weak, with a *K*_D_ 356 μM [CI 138, 428 μM]. No binding was observed to the medium chain fatty acid decanoate (Supplementary Figure 2), suggesting this was a false positive; the positive response observed in the FTS assays may have been due to aggregation and dye-binding of the hydrophobic decanoate molecule. Overall, these results suggest that the Cache domain of PscD can bind with reasonable affinity to C_2_ and C_3_ carboxylates, and that it has the highest affinity towards the two-carbon carboxylates glycolate and acetate.

### *Psa* chemotaxis towards C_2_ and C_3_ carboxylates only partially correlates with their utilisation as nutrient sources

**Figure 3:**
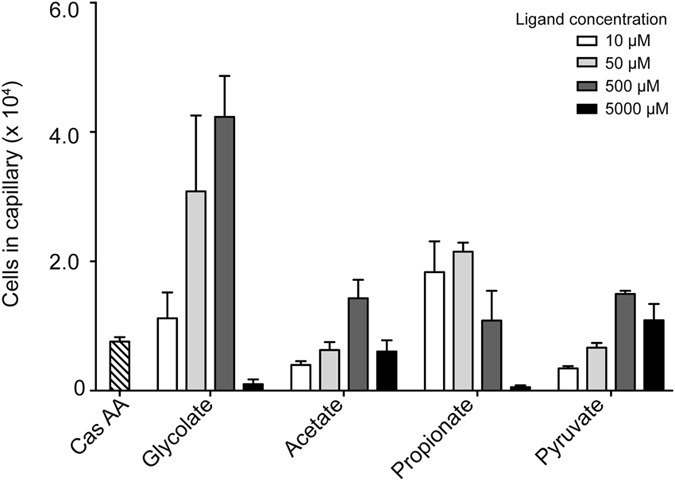
The chemotactic response of *Psa* towards the four carboxylate ligands recognised by PscD-SD. The chemotactic response of *Psa* to various concentrations of potential chemoeffectors were measured by a quantitative capillary chemotaxis assay. The data have been normalised to a buffer only control by subtracting the average number of cells that accumulated in capillaries containing only the chemotaxis buffer. Also shown is the positive control response to casamino acids (Cas AA). The data represent the means and standard errors of the mean of three independent assays (n = 3).

The chemotactic response of *Psa* towards glycolate, acetate, propionate and pyruvate was tested with quantitative capillary assays. In these assays, the response is quantified by comparing the numbers of cells that navigate into a capillary containing a potential chemoattractant, versus the number of cells that accumulate in a capillary containing only buffer. The chemotactic response was measured over a range of concentrations for each ligand (10 μM to 5000 μM). *Psa* showed positive chemotaxis towards all four ligands (Fig. 3). In all cases, the response was concentration dependent. Chemoattraction was observed at even the lowest concentration tested (10 μM), indicating that *Psa* is strongly attracted towards these ligands. However, the most effective concentration was in the range of 50 μM to 500 μM of chemoattractant. At higher concentrations (5000 μM) the strength of the chemoattraction decreased, which may be due to induced toxicity or the effect of lowering pH beyond what the bacterium can tolerate. Overall, glycolate was the strongest attractant, with >2-fold more cells per capillary than the other ligands tested.

While one important function of chemotaxis is to sense nutrients, bacteria also display chemotaxis towards non-metabolisable chemicals such as environmental and/or host signals^24^,^25^. To assess whether the identified chemoattractants could be utilised as nutrient sources by *Psa*, we conducted growth experiments using Biolog PM plates 1 and 2 with each chemoattractant as the sole carbon source. The results showed that acetate and pyruvate are metabolisable, growth-supporting carbon sources for *Psa*. In contrast, *Psa* was not able to utilise glycolate or propionate as a carbon source (full results shown in Supplementary Figure 3), despite these compounds acting as chemoattractants.

### Overall structure and ligand binding site of PscD-SD

To shed light on the molecular basis for recognition of C_2_ and C_3_ carboxylates by PscD, we determined the three-dimensional structure of PscD-SD (residues 31–188) using X-ray crystallography.

**Figure 4:**
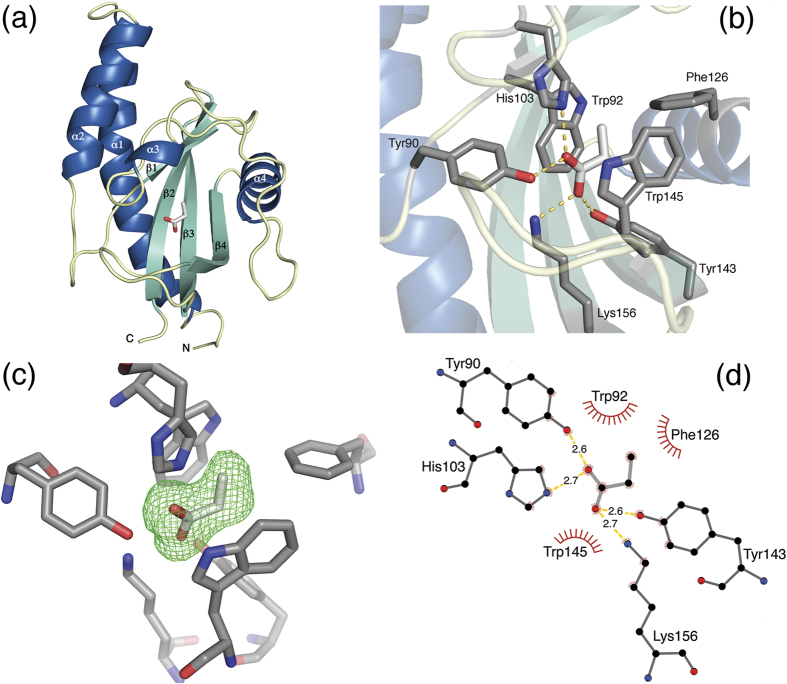
X-ray crystal structure of the Cache sensor domain of PscD (PDB ID 5G4Z). (**a**) Ribbon diagram of the overall structural fold of PscD-SD with propionate bound. The α helices are shown in blue, β strands in green, and loops in yellow. Propionate is shown as sticks, with the carbon atoms coloured light grey, and oxygen atoms coloured red. (**b**) The ligand binding site of PscD-SD including hydrogen bonds (dashed yellow lines) between propionate and the surrounding amino acid residues. Amino acid side chains are shown in sticks representation with carbon atoms in dark grey; propionate is shown with carbon atoms in light grey. Oxygen and nitrogen atoms are coluored red and blue, respectively. (**c**) Difference density (green wireframe) seen after removing the propionate ligand and map calculation. m*F*_o_-D*F*_c_ difference maps (3σ level) were calculated using the final refined model after the propionate ligand was removed; the orientation is approximately the same as in panel b with the propionate shown as a stick model. (**d**) Diagram representation of the PscD binding site. Hydrogen bonds are represented by dashed lines (yellow) and the bond distances in Ångstroms are marked on the figure. Hydrophobic interactions are represented as red arcs with spikes. Panel (d) was drawn using LigPlot+^43^.

Two structures were determined: one from crystals prepared from the protein alone (PDB:5G4Y) and the other following the soaking crystals with the C_3_-ligand propionate (PDB:5G4Z). Both structures were refined to approximately 2 Å; the crystallographic data and refinement statistics are presented in Table 1. Residues 31–178 are well ordered and are included in the final models, whereas the N-terminal His_6_ tag and TEV protease site are disordered, and are distant from the ligand binding site. The Cache domain of PscD consists of an N-terminal helix-loop-helix, followed by an antiparallel five strand β-sheet, which is surrounded by additional helices on either side (Fig. 4A). The long N-terminal alpha helix is characteristic of Cache domains, as compared to similar folds such as PAS or GAF^7^. Our two structures overlay with an RMSD of 0.1 Å over all Cα atoms, indicating that they are effectively identical. In both structures, there is clear electron density corresponding to a small molecule present in the canonical ligand binding site. Propionate models very well into the electron density of both structures, whereas alternative ligands (*e.g*. acetate, glycerol) could not be reasonably modelled into the observed density. Propionate was not present in the purification or crystallisation buffers, indicating that it was scavenged from the cells and/or medium and supporting the idea that it is a physiologically relevant ligand for PscD.

The ligand binding site is an open and flexible region of the Cache domain, formed by a β-sheet, large loops and a short α-helix. Propionate is bound within a pocket formed by β-strand 1 (Trp92 – Asp95), α-helices 3 (Pro107 – Leu110) and 4 (Val125 – Lys136) interspersed by long loop regions and β-strands 2–4 (Ala138 – Trp145, Val154 – Phe163 and Gly167 – Tyr175 respectively), which form a β-sheet. The carboxyl oxygen atoms of propionate form hydrogen bonds with four residues: Tyr90, His103, Tyr143 and Lys156 (Fig. 4B,C) and the ligand is sandwiched between two tryptophan residues, Trp92 and Trp145, giving the impression of being ‘flat-packed’ with distances of 3.5 to 4.2 Å (Supplementary Figure 4). The hydrophobic residue Phe126 faces the methyl group of propionate, forming a back-end wall of the pocket.

### Structural and sequence comparisons

A search using the Dali server^26^ revealed a handful of Cache domains with high structural similarity to PscD-SD. The top hit was a Cache sensor domain of an uncharacterised chemoreceptor from *Anaeromyxobacter dehalogenans* (Adeh_3718, PDB entry 4K08; Z score 25; 42% identity)^27^. More distant structural homologues include an uncharacterised sensor domain from *Vibrio parahaemolyticus* (PDB entry 4EXO; Z score 21; 23% identity) and TlpB from *Helicobacter pylori* (PDB entries 3UB8-9; Z score ~18; 21% identity)^28^. With the exception of TlpB, which was crystallised with urea in the binding site, the crystallisation conditions of the other sensor domains suggest that they also bind C_2_ and C_3_ carboxylates: Adeh_3718 crystallised with acetate in its binding site, while the *V. parahaemolyticus* SD binding site is occupied by pyruvate.

**Figure 5:**
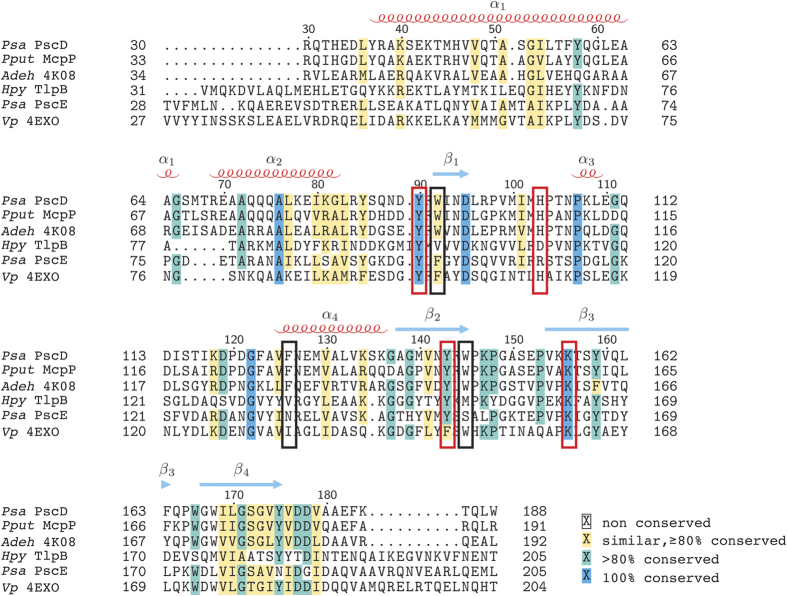
Multiple sequence alignment of PscD-SD with other known single Cache chemoreceptor sensor domains. The alignment was performed using MUSCLE with shading in T_E_XShade by L^A^T_E_X 2_ε_ using shading mode similar. Sequences are labelled by species abbreviation and either protein name or PDB code. Secondary structural elements of PscD-SD are mapped onto the alignment with alpha-helices (labelled α_1_-α_4_) indicated by red coils and beta-strands (labelled β_1_-β_4_) indicated by blue arrows. Red boxes indicate amino acids that form polar contacts with the ligands and black boxes indicate other amino acids in the binding-site.

A sequence alignment of PscD-SD with these and other characterised Cache domains was used to identify amino acid residues involved in determining ligand specificity (Fig. 5). The sequence alignment includes a recently identified chemoreceptor sensor domain from *P. putida* which has been shown to bind C_2_ and C_3_ carboxylates (*i.e*. acetate, pyruvate, propionate, and L-lactate), though its structure has not been determined^29^. The binding pockets of the sensor domains that bind C_2_ and C_3_ carboxylates are highly conserved. All seven amino acid residues that make contact with the propionate ligand (via polar or hydrophobic interactions) in the structure of PscD-SD (Fig. 5) are identical between the PscD-SD, McpP-SD, and Adeh_3718 sequences (Fig. 5). The sensor domain from *V. parahaemolytics* shares three of the four key amino acids involved in forming hydrogen bonds to the ligand (equivalent to Tyr90, His103 and Tyr143 in PscD), but makes an additional hydrogen to the β-carbonyl of pyruvate via a tyrosine residue (Tyr70) not present in PscD (the equivalent residue in the PscD structure is Asn94) (Supplementary Figure 6). There is also much greater variation in the non-carboxylate binding residues of TlpB and the sensor domain from *V. parahaemolyticus* (Fig. 5, Supplementary Figure 6). TlpB is the only single Cache domain included that does not bind carboxylate ligands. Perhaps unsurprisingly, there is little sequence or structural similarity between the binding pocket of this sensor domain and the others (Fig. 5, Supplementary Figure 6). Furthermore, some of the residues that are conserved in the sequence alignment are not equivalent in the corresponding structures. For example, one of the tyrosine residues (Tyr90) involved in ligand binding in PscD is identical in the TlpB sequence, but examination of the TlpB structure reveals that this residue does not participate in binding urea.

## Discussion

Here we have reported a combination of ligand-binding, phenotypic and X-ray crystallographic studies of a single Cache sensor domain. In the first part of this study, we utilised the high-throughput FTS assay that we developed previously^16^ to identify potential ligands of a *Psa* chemoreceptor that we have designated PscD. Our initial screen of 190 ligands identified nine potential hits. Building on our previous work (with a single Biolog plate; 95 ligands), we confirmed that FTS assays are a fast and inexpensive primary screening method, although the present study also emphasises that it is essential to cross-validate hits by other binding or functional assays. Four of the nine candidate ligands (all C_2_ or C_3_ carboxylates: glycolate, acetate, propionate, and pyruvate) were found to bind PscD-SD directly, as determined using ITC. More generally, our approach also illustrates the advantage of using Biolog plates as the source of a large, diverse and biologically-relevant set of ligands, as they enable a standardised approach for comparative studies. For example, *Psa* PscD, *P. putida* McpP, and Adeh_3718 all bind acetate (and the PscD and McpP sensor domains have comparable affinities of 31 μM and 34 μM^29^ respectively). However, the PscD has the highest affinity (and chemoattraction) for glycolate – which has not been tested as a potential ligand with the other Cache-type domains. The lack of a standardised testing protocol therefore makes it unclear whether glycolate is a common function of single Cache domains, or if PscD-SD is atypical in this regard.

The ligand binding repertoire of PscD hints at the potential biological role of carboxylate sensing for *Psa*. Chemotaxis plays an important role in the interactions between plants and bacteria. Plants exude a diverse array of molecules including sugars, amino acids, organic acids, aromatics, and secondary metabolites, all of which may be chemoattractants (but not necessarily nutrients) for plant-associated bacteria^30^. *Psa* is a bacterial foliar pathogen that is able to grow and survive on leaf surfaces before invading the host plant (kiwifruit, *Actinidia* spp.) through pre-existing openings such as stomata, lenticles, wounds and lesions^9^,^10^. The leaf surface, or phyllosphere, is a competitive and nutrient limited environment^31^. Critically, the distribution of nutrients is not homogeneous: nutrients are abundant at particular places on the leaf surface (such as stomata) due to localised accumulation at the site of exudation. This patchy distribution of nutrients in the phyllosphere poses different survival challenges compared with the nutrient-dense habitat of, for example, an enteric bacterium. We have shown that the four carboxylate ligands identified for PscD-SD are also chemoattractants for *Psa*. Two of the ligands of PscD-SD, acetate and pyruvate, are utilisable carbon and energy sources for *Psa*, feeding into the tricarboxylic acid cycle. Thus one role of PscD may be to guide *Psa* towards scarce nutrients. However, our results also show that *Psa* cannot utilise glycolate or propionate as a carbon source. It may be instead that *Psa* detects glycolate and propionate as ‘guidepost’ molecules for movement across the leaf surface towards stomata, playing a role in bacterial infiltration of the host plant. Consistent with this idea, the exudation of glycolate has been linked to stomatal opening (reviewed by^17^). Further research, investigating the role of this and other chemoreceptor sensor domains in *Psa*, will provide much needed insights into the role of chemotaxis in host recognition and colonisation.

Examination of the PscD-SD crystal structure provides clues as to the structural basis of its relative binding affinities towards C_2_ and C_3_ carboxylate ligands. PscD-SD has the highest affinities for the smaller C_2_ carboxylates, glycolate and acetate (*K*_*D*_ values of 23 μM and 31 μM respectively) with binding stabilised by the four hydrogen bond interactions with Tyr90, His103, Tyr143 and Lys156. Though glycolate potentially has an additional hydroxyl group available for hydrogen bonding (as compared to acetate) it is directed towards either Trp92 or Trp145 depending on its orientation, and this hydroxyl oxygen is predicted to be too distant (~3.5 Å) from the side-chain nitrogen atoms of either Trp92 or Trp145. PscD-SD exhibits comparatively lower affinities for the C_3_ ligands propionate and pyruvate (*K*_*D*_ values of 101 μM and 356 μM respectively). The electron density for propionate (Fig. 4 and Supplementary Figure 7) indicates that the Cγ positioned away from Tyr143, minimising the potential steric clash. This restriction in orientation may result in a lower affinity relative to the C_2_ ligands, which can be comfortably accommodated in either orientation of the carboxylate plane. The hydroxyl of pyruvate is predicted to form an additional hydrogen bond through His103, which would also restrict its orientation in the binding site. However, this also positions the Cγ methyl group of pyruvate within less than 2.5 Ă of both Met129 and Tyr143, which likely creates significant steric constraints resulting in a much lower binding affinity. Overall, the bulky hydrophobic residues that line the binding pocket appear to prevent the binding of larger (C_4_ and larger) carboxylates. This is supported by the *in vitro* binding data, which show an increase in *K*_*D*_ (decreasing affinity) with the length of the carbon chain, and is also consistent with the results of our high-throughput FTS screening, where larger C_4_ and C_5_ carboxylates did not stabilise the protein (ΔT_m_ < 1 °C, Fig. 1, Supplementary Table 1).

Currently, there are 822 sequences identified in the Pfam database that are predicted to have single Cache-like sensor domains^18^. Of these, over half (>500) are predicted to occur within chemoreceptors (*i.e*. they are coupled to an MCP signalling domain). Based on our current knowledge, can we make any predictions about their specificity? Overall, a sequence alignment of the five single Cache domains with known ligands, suggests that three residues (Tyr90, His103 and Lys156 in PscD) are critical for the formation of hydrogen bonds with the oxygen atoms of the carboxyl group of the ligand. An examination of the seed alignment for the single Cache family model (29 sequences) shows that these three residues are highly conserved (Supplementary Figure 8), suggesting that other single-Cache containing chemoreceptors may also detect ligands with carboxylate functional groups. However, our structure suggests that the position of the flanking hydrophobic residues dictate the sizes of the carboxylate ligands accommodated in the binding pocket - and a survey of sequences shows that the residues at these positions are far less conserved. Thus, it appears that single Cache domains are relatively specialised for the detection of carboxylate ligands. PscE, likely also fits into this model. Two of the three carboxylate binding amino acids are conserved in PscE (His103 in PscD is replaced with an Arg in PscE, Fig. 5). This substitution, combined with the overall low sequence identity between the PscD and PscE sensor domains, suggests that PscE may bind a different range of carboxylates. However, further work exploring the sequence diversity (and therefore potential functional diversity) of this widespread class of sensor domains is required. In addition, it is worth noting that even when sensor domains have highly conserved binding pockets, and bind the same general class of ligands, their affinities for specific ligands may be different. For example, seven of the seven binding pocket residues are identical between PscD and McpP. However, McpP has a relatively broad specificity for carboxylates, with similar affinities for both C_2_ and C_3_ carboxylates, including pyruvate (*K*_D_s ~ 30 μM)^29^. In contrast, PscD has the highest affinity towards the C_2_ carboxylates, and binds pyruvate quite weakly (*K*_D_ = 356 μM). These differences highlight the need for experimental verification for predicted functions, even in cases where sequences are conserved.

In summary, the structure of PscD-SD adds to the small (but growing) number of characterised single-Cache domains used as sensors in many chemoreceptors and other signal transducers. These results provide insights into their specificity for small carboxylate ligands, and hints at possible roles for carboxylate sensing for the survival of plant pathogens in the phyllosphere.

## Materials and Methods

### Materials

Molecular biology enzymes were purchased from New England BioLabs (Ipswich, MA, USA). Oligonucleotides were from Integrated DNA Technologies (Coralville, IA, USA). Growth media were Formedium (Hunstanton, Norfolk, UK). Talon metal affinity resin was from Clontech (Mountain View, CA). Benzonase nuclease, protease inhibitor cocktail, chicken egg white lysozyme, and all other chemicals were from Sigma Chemical Co. (St. Louis, MO, USA) unless otherwise noted. Phenotype microarray plates were from Biolog Inc. (Hayward, CA, USA).

### Bioinformatics analyses and cloning of sensor domains

The annoted genome sequence of *Psa* ICMP 18884 (accession CP011972^15^) was used to identify putative chemoreceptor genes. Predicted Cache-containing chemoreceptors were identified using Pfam 29.0^18^, and their transmembrane regions were predicted using TOPCONS^19^. Two chemoreceptors were predicted to encode single Cache domains: accession AKT31526 encoded by locus tag IYO_018780, which was designated PscD; and AKT30128 encoded by locus tag IYO_011445, designated PscE. The DNA fragments encoding predicted Cache-like sensor domains were amplified from genomic DNA using Phusion polymerase. The DNA fragment encoding the predicted sensor domain of PscD (Gln31-Trp188) was amplified using with the primers 5′-CAGCATATGCAGACTCATGAAGACCTGTATCG-3′ and 5′-CAGTCTCGAGTTACCACAGCTGAGTCTTGAACTCTG-3′. The DNA fragment encoding the predicted sensor domain of PscE (Met31-Tyr206) was amplified using the primers 5′-CAGCATATGGTCAGCGATACGCGAGAG-3′ and 5′-CAGTCTCGAG TTACGCTTCAACGTTCTGCCT-3′. Several additional truncations of the PscE sensor domain (Val40-Ala199, Val40-Asp187) were constructed in a similar fashion. Amplified DNA was cloned into a pET28(a+) expression vector to generate N-terminal his-tagged fusion proteins, and the constructs were verified by DNA sequencing. The resulting plasmids (pET28-PscD-SD and pET28-PscE-SD) were used to transform the expression host strain *E. coli* BL21 GOLD (DE3).

### Protein expression and purification

*E. coli* cultures were grown in phosphate buffered Terrific Broth (ForMedium, Hunstanton, Norfolk, UK), with 30 μg.mL^−1^ kanamycin sulfate (PanReac AppliChem, Darmstadt, Germany), at 37 °C to an OD_600nm_ = 0.6, before protein expression was induced with 0.5 mM isopropyl β-D-1 thiogalactopyranoside. The cultures were cooled to 18 °C and cultured overnight. After cultivation, cells were harvested by centrifugation and were resuspended in lysis buffer (50 mM potassium phosphate pH 7.0, 300 mM sodium chloride and 10% glycerol) with 5 U.mL^−1^ Benzonase nuclease, 4 μL.mL^−1^ protease inhibitor cocktail, and 0.5 mg.mL^−1^ egg white lysozyme. Cells were lysed by sonication on ice. Following lysis, insoluble protein and cellular debris was pelleted by centrifugation, and the His_6_-tagged recombinant protein was purified from the soluble cell lysate using TALON Metal Affinity Resin (Clontech). The proteins were further purified using size-exclusion chromatography. Pooled elution fractions were injected onto a 16/60 S200 Superdex column (GE Healthcare, Chicago, IL, USA), pre-equilibrated with storage buffer (50 mM sodium phosphate pH 7.0, 200 mM sodium chloride and 10% glycerol). The protein fractions of interest were pooled and concentrated using Amicon Ultra 15 centrifugal concentrators with a molecular weight cut-off of 10 kDa (EMD Millipore, Billerica, MA, USA). Protein purity was assessed by SDS-PAGE and protein concentrations were quantified by measuring *A*_280_ (ε = 8370 M^−1^ cm^−1^ for PscD-SD, calculated according to ref. 32).

### High-throughput fluorescent thermal shift screening for ligand binding

Fluorescent thermal shift (FTS) assays were conducted as reported previously^16^. PscD-SD was screened for potential ligands against Biolog Phenotype Microarray (PM) plates 1 and 2 (Biolog, Hayward, CA, USA). For initial qualitative, high-throughput screening, ligands were prepared by dissolving Biolog PM compounds in 50 μL of water (each) to obtain a final concentration of approximately 10–20 mM (the exact concentrations vary from well-to-well). Screens were set up in Lightcycler 480 multiwell plates (Roche Diagnostics, Auckland, NZ) with 20 μM protein, a 5x concentration of SYPRO orange (Life Technologies, Carlsbad, CA, USA) and 5 μL of the test compound (*i.e*. resuspended Biolog ligands) added to each well. The plate was covered with Lightcycler 480 sealing film (Roche Diagnostics) and fluorescence signal was monitored as the temperature was increased from 20 °C to 80 °C at a rate of 1.2 °C.min^−1^ in a Lightcycler 480 (Roche Diagnostics). Initial hits were further tested using known concentrations (0, 1, 2, 5 and 10 mM) of each potential ligand to confirm binding. The ligands were prepared as 10x stocks, 2 μL were added to each well, and the experiments were conducted as described above. The first derivative values (−dF/dt) from the raw fluorescence data were used to determine the melting temperature (T_m_). Three biological replicates (from independently cultured and purified batches of protein) were performed for each plate screened.

### Isothermal titration calorimetry

All experiments were carried out using a MicroCal VP-ITC (Malvern Instruments, Malvern, UK). Proteins were dialysed overnight in storage buffer and then diluted to a concentration of 30 μM; ligands were prepared to a concentration of 900 μM using dialysis buffer, in order to minimise the heat effects of dilution during the injection. Ligands were titrated into the protein sample at 24 °C using 28 injections of 10 μL each, with 320 s spacing between injections. Attempts to increase the concentration of protein to obtain a higher *c*-value in the titration experiments resulted in protein aggregation. Baseline data were measured by titration into buffer and subtracted from experimental data. Data were analysed using NITPIC^33^ and SEDPHAT^34^. ITC was performed with at least two biological replicates (from independently cultured and purified batches of protein) for each protein and ligand combination tested. The biological replicates were analysed using global fitting in SEDPHAT using the simple 1:1 binding model [A + B → AB]. In this global fitting, the reaction stoichiometry is fixed in the reaction model and instead separate parameters allow for concentration errors and/or incompetent fractions of material^35^.

### Quantitative *in vivo* chemotaxis assay

Chemotaxis assays of *Psa* strain NZ-V13 were performed essentially as described previously^16^. Briefly, 20 mL of tryptone broth (1% tryptone, 0.5% sodium chloride) was inoculated by taking a stab from the outer edge of the growth ring of passage number 4 and incubated for 18 h at 28 °C, until an OD_600nm_ = 0.3 was reached. Cells were harvested by centrifugation at 4000 *g* for 5 min at 20 °C. The supernatant was removed and cells were resuspended in chemotaxis buffer (10 mM HEPES pH 7.0) to an OD_600nm_ = 0.2 and incubated at room temperature for 2 h. Ligands were dissolved and diluted into chemotaxis buffer to concentrations of 10 μM, 50 μM, 500 μM and 5000 μM. Microcaps 1-μL glass capillaries (Drummond Scientific, PA, USA) were flame-sealed at one end and incubated for 2 h in 100 μL of ligand solution then transferred into 100 μL of *Psa* cell suspension for 20 min. The external surface of the capillary was rinsed with distilled water and the contents were expelled into 1 mL of chemotaxis buffer. Dilutions were plated onto Luria Bertani (LB) agar and the plates were incubated for 48 h at 28 °C. Colonies were counted to determine the number of cells per capillary, which was normalised to a chemotaxis buffer only control. Six replicates of each ligand and concentration combination were counted from three independent experiments.

### Nutrient utilisation profiling

The ability of *Psa* to utilise various carbon compounds was investigated using Phenotype Microarray (PM) plates^36^. A starter culture of *Psa* was grown overnight in LB broth at 28 °C. In the morning a 500 μL aliquot from the overnight culture was used to inoculate 20 mL LB. Once the culture reached an OD_600_ of 0.7, cells were harvested *via* centrifugation at 2,000 × g for 15 min and resuspended in sterile milliQ water to an OD_600_ of 0.4. The cell suspension was incubated for 1 h at room temperature to deplete nutrient reserves of the cells. Cells were inoculated into Inoculation Fluid (IF-0) and Dye Mix A (Biolog Inc.), from which 100 μL was added to the each of the 96 wells of both PM1A and 2B plates. The plates were incubated at 28 °C for 48 h. The cells were monitored for growth visually, based on the colour change of the tetrazolium indicator dye.

### Crystallisation

Protein at 12 mg.mL^−1^ in storage buffer was set up in the 1-click-screening protocol at CSIRO’s Collaborative Crystallisation Centre (384 conditions: shotgun screen at both 20 °C and 8 °C, as well as PACT and PS gradient screens at 20 °C, with droplets consisting of 150 nL protein solution and 150 nL reservoir, set up as sitting drops against a reservoir of 50 μL in SD-2 crystallisation plates (Molecular Dimensions, UK); details of the screens can be found at http://c6.csiro.au). Under these conditions, no obvious leads were obtained. The protein was then dialysed into a formulation of 50 mM bis-tris chloride pH 6.5, 50 mM sodium chloride, which had been shown by FTS to give a significant increase in the melting temperature of the protein (ΔT_m_ ~ 13 °C). Protein at 8.5 mg.mL^−1^ in this buffer was screened using the same one-click screening protocol. The best lead from this screen (shotgun screen, condition H5) contained 30% PEG 4000. This was used as the basis for additive screening, using the HT additive screen (Hampton Research), and a number of additives gave large single hexagonal bipyramidal crystals at 20 °C.

### X-ray diffraction, data collection and processing

A crystal grown at 20 °C from 8.5 mg.mL^−1^ protein buffered with 50 mM bis-tris chloride pH 6.5, 50 mM sodium chloride and precipitant conditions of 27% PEG 4000 with 0.1 M proline was cryo-protected with well solution fortified with 20% glycerol and flash-cooled in liquid nitrogen. A full data set of 180 one degree oscillations was obtained from the MX2 beamline of the Australian Synchrotron with the data extending beyond 2 Å (see Table 1 statistics). The crystal structure was solved by molecular replacement using PDB entry 4K08 as the model. The data were first integrated with XDS^37^, scaled with the program Aimless^38^, and the structure was phased using Phaser^39^. The model was prepared using Chainsaw^40^. The structure was then rebuilt manually using Coot^41^ and refined with the program REFMAC5^42^. Coordinates have been deposited in the PDB with the ID code 5G4Y. The second crystal was also grown at 20 °C from 8.5 mg.mL^−1^ protein buffered with 50 mM bis-tris chloride pH 6.5, 50 mM sodium chloride but with precipitant conditions of 27% PEG 4000 and 3% sucrose. Propionate was soaked into the crystal by the addition of 40 mM neutralised propionic acid and 1 μL of well solution to the crystal drop, the well was then resealed and left for 24 hours. Propionate was chosen for the soaking experiments as it was hoped that a C_3_ ligand would provide a stronger electron density signal than the smalller C_2_ ligands acetate or glycolate. In addition, we were interested in understanding the basis of C_3_ ligand accomodation in the binding site in relation to differences in binding affinintes observed between the C_2_ and C_3_ ligands. The crystal was subsequently cryo-protected with 20% glycerol prior to flash-cooling in liquid nitrogen. Data collection, processing and structure solution were as before, but the structure was solved using Phaser with the previously derived ‘native’ model above. The coordinates of the propionate soaked structure were deposited in the PDB with the ID code 5G4Z.

## Additional Information

**Accession codes**: Coordinates have been deposited at the Protein Data Bank (PDB) with accession numbers 5G4Y (native) and 5G4Z (propionate soaked).

**How to cite this article**: Brewster, J. L. *et al*. Structural basis for ligand recognition by a Cache chemosensory domain that mediates carboxylate sensing in *Pseudomonas syringae*. *Sci. Rep*. **6**, 35198; doi: 10.1038/srep35198 (2016).

## Supplementary Material

Supplementary Information

## Figures and Tables

**Table 1:** Crystallographic parameters and refinement statistics.

PDB	5G4Y	5G4Z^1^
Space group	P6_4_	P6_4_
Cell dimensions		
*a*, *b*, *c* (Å)	74.3, 74.3, 75.1	73.4, 73.4, 74.9
α, β, γ (ϒ)	90, 90, 120	90, 90, 120
Resolution (Å)	48.8–2.00 (2.05–2.00)	48.5–1.98 (2.03–1.98)
*R*_merge_	0.116 (0.810)	0.098 (0.918)
*R*_pim_	0.036 (0.257)	0.031 (0.285)
*CC1/2*	0.998 (0.938)	0.999 (0.940)
*I*/σ*I*	14.2 (2.9)	18.7 (3.3)
Completeness (%)	99.8 (97.8)	100 (100)
Redundancy	11.2 (10.8)	11.1 (11.2)
Refinement		
Resolution (Å)	48.8–2.00	48.5–1.98
Unique reflections	15,217	15,189
*R*_work_/*R*_free_ (%)	16.2/18.2	16.5/17.6
No. atoms	1,260	1,266
Protein	1,155	1,192
Ligand	5	5
Water	80	59
*B*-factors (Å^2^)	36.6	37.3
Protein	36.2	36.8
Ligand	29.4	30.5
Water	41.7	41.1
R.m.s. deviations		
Bond lengths (Å)	0.017	0.020
Bond angles (ϒ)	1.794	1.972
Ramachandran statistics		
Favoured (%)	98.6	98.0
Allowed (%)	1.4	2.0
Outliers (%)	0	0

Values in parentheses are for the highest-resolution shell.

^1^Structure resulting from soaking PscD-SD crystals with propionate.
