# Supplementary material for: Structural basis for ligand recognition by a Cache chemosensory domain that mediates carboxylate sensing in Pseudomonas syringae
Source: Sci Rep. 2016 Oct 13;6:35198. doi: 10.1038/srep35198 (PMC5062169; doi:10.1038/srep35198)
Supplement: Supplementary Information [file srep35198-s1.pdf]

Supplementary Information

Structural basis for ligand recognition by a Cache chemosensory domain that mediates carboxylate sensing in *Pseudomonas syringae*

Authors

Jodi L. Brewster[1], James L.O. McKellar[1], Thomas J. Finn[1], Janet Newman[2], Thomas S. Peat[2], Monica L. Gerth[1*]

Supplementary Figure 1

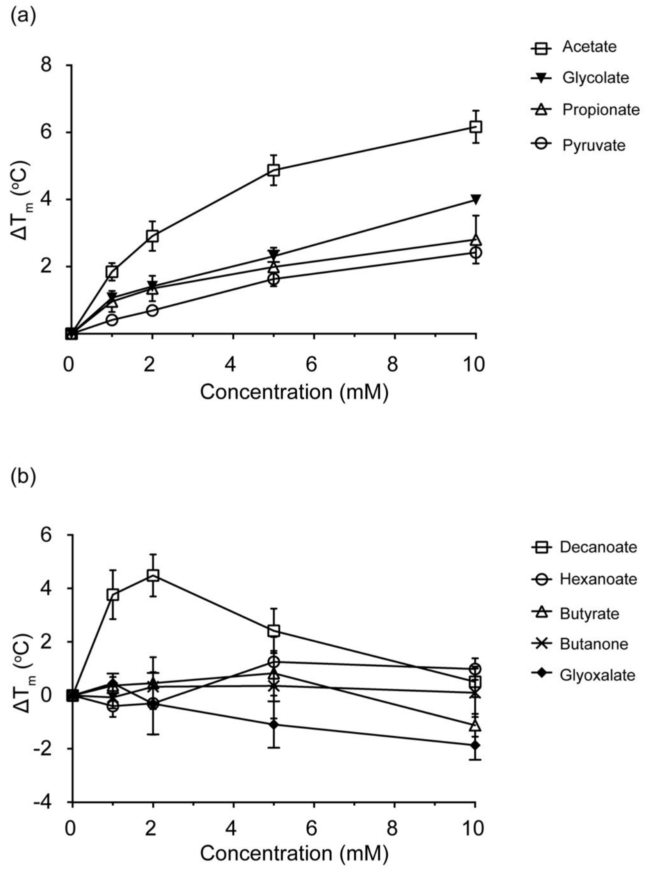

Figure S1. Re-screening PscD-SD with known, increasing ligand concentrations to assess potential binding. Results of FTS assays of: (a) Rescreening potential hits from Biolog Plate PM1. (b) Rescreening potential hits from Biolog Plate PM2. Data are the means and standard deviations from three experiments. If not visible, the error bars are contained within the symbol.

Supplementary Figure 2

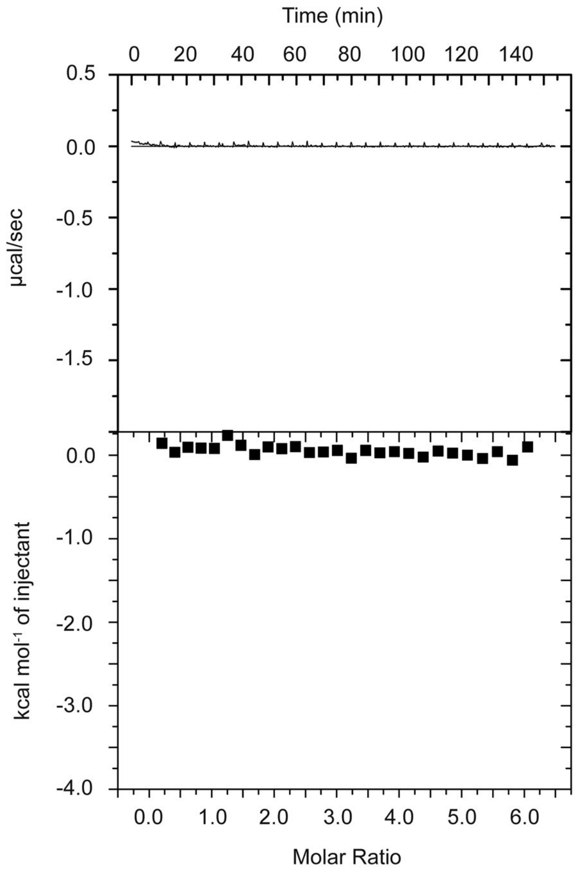

Figure S2. Representative isothermal titration calorimetry plot of the *Psa* PscD-SD with decanoate. Titration of 30 µM of PscD-SD with 0.9 mM decanote showed no significant heat signals that would indicate binding.

Supplementary Figure 3

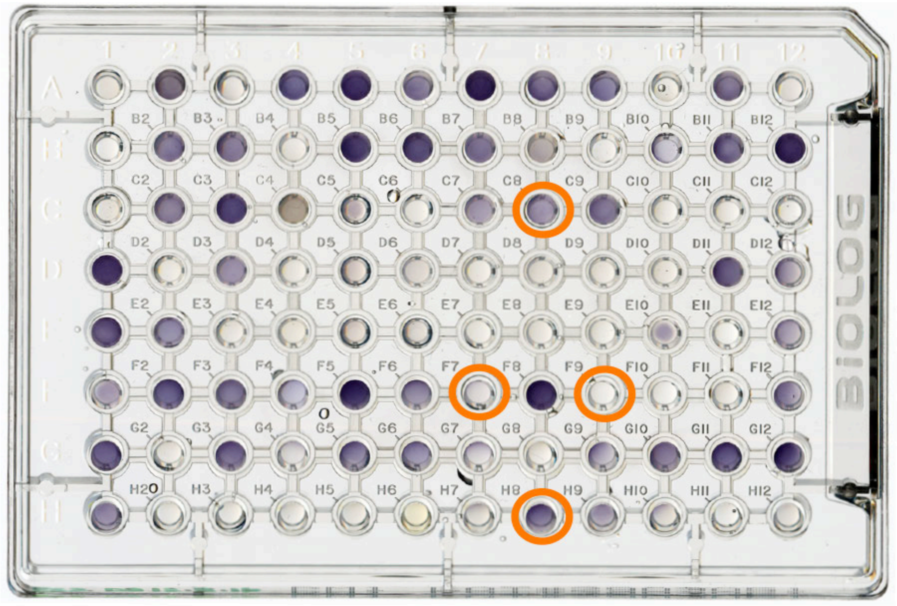

Figure S3. Phenotype microarray (PM) screening for carbon source utilization. The growth of *Pseudomonas syringae* pv. actinidae (*Psa)* was investigated using PM plate 1. Growth is indicated by purple color development in the wells due to the presence of a tetrazolium indicator dye. The four compounds identified as ligands of PscD-SD are in the following wells (highlighted with orange circles): C8, acetate; F7, propionate; F9, glycolate; and H8, pyruvate.

Supplementary Figure 4.

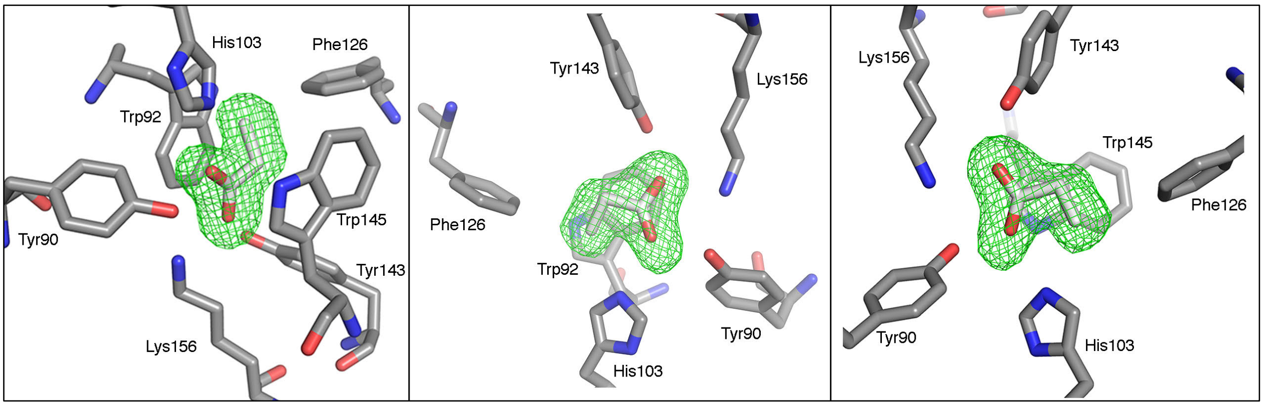

Figure S4. Three different views (orientations) of a mF_o-DF_c omit map where the ligand (propionate) has been removed from the model prior to generating the map. The green wire density is shown at 3σ. Panel (a) has all key residues shown; panel (b) has Trp145 omitted and panel (c) has Trp92 omitted for clarity.

Supplementary Figure 5.

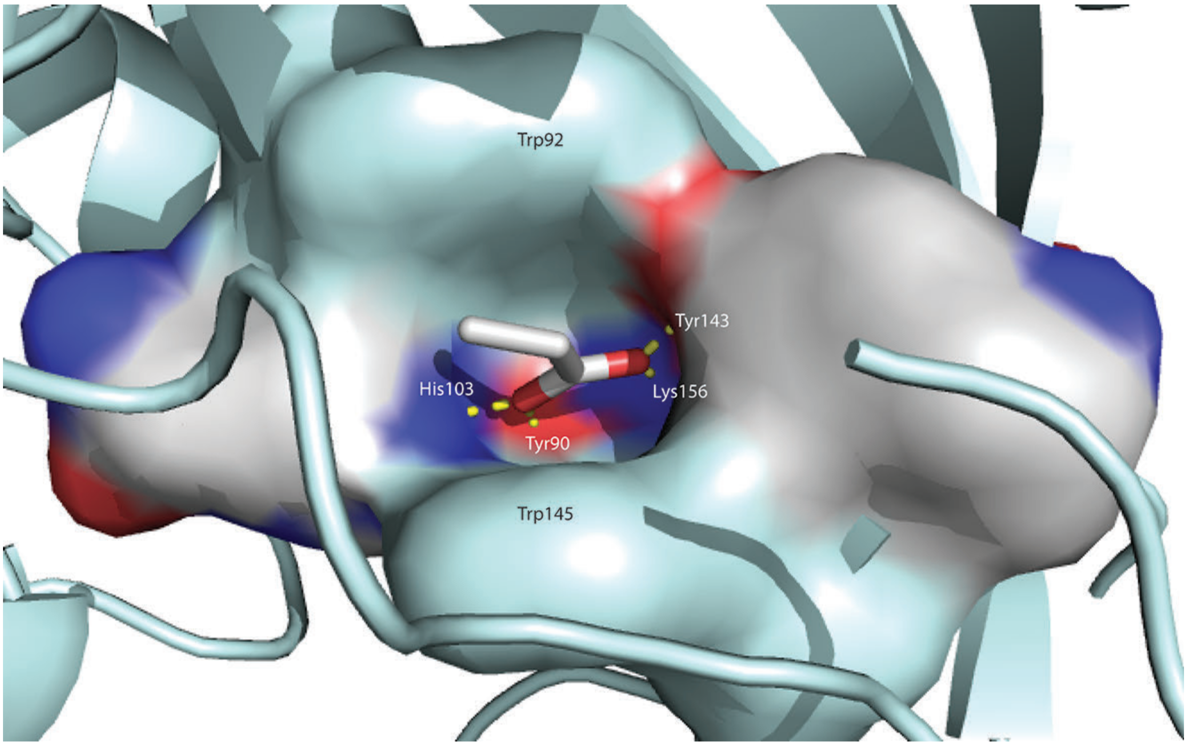

Figure S5. Three-dimensional surface model of propionate in the PscD-SD ligand binding site. The four amino acids that hydrogen bond to propionate; Tyr90, His103, Tyr143, Lys156, are colored by atom with carbon in grey, nitrogen in blue and oxygen in red. Residues Val125 – Met129, which includes hydrophobic binding site residue Phe126, block the view of the pocket and have been omitted for clarity.

Supplementary Figure 6.

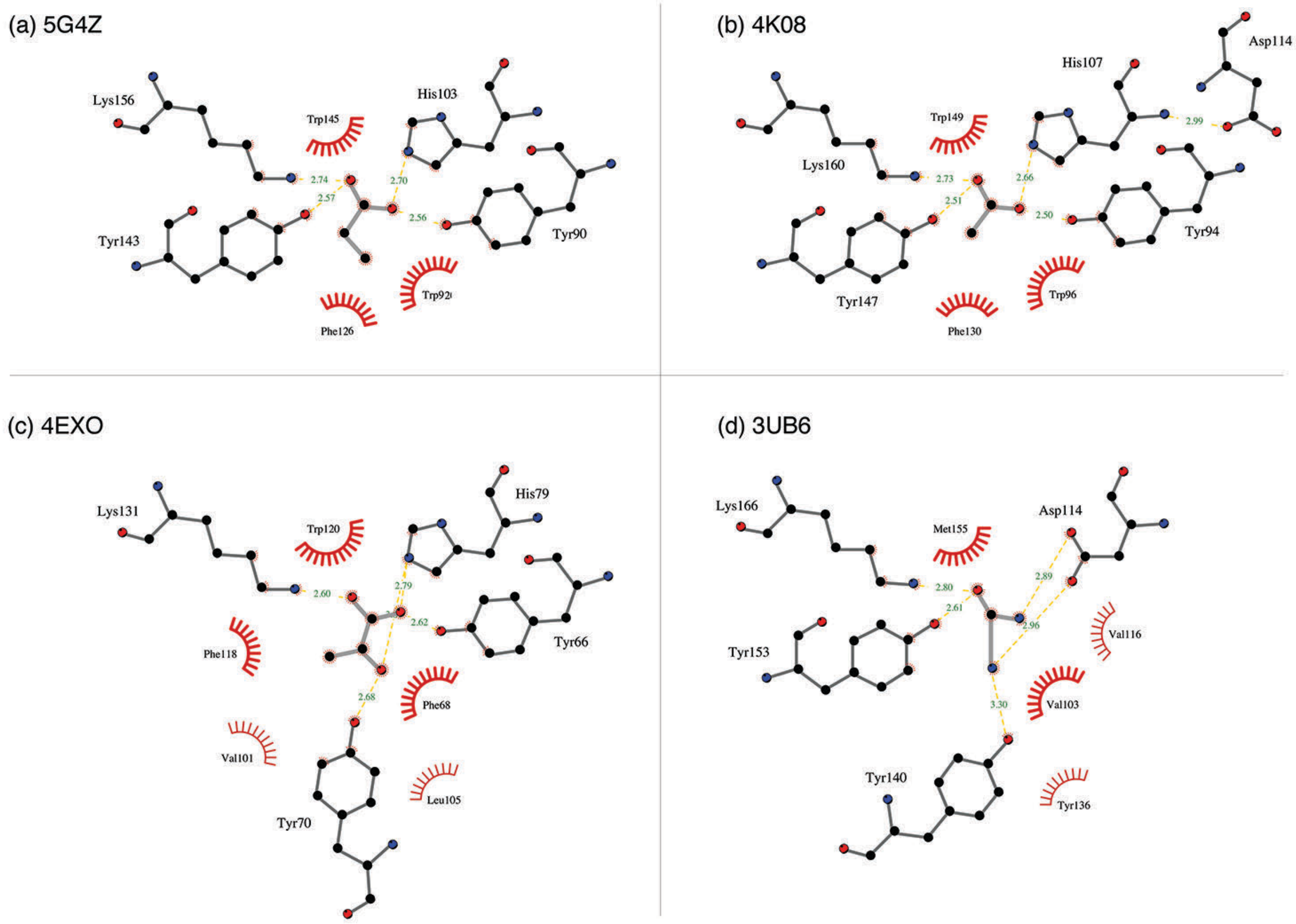

Figure S6. Comparisons of the PscD-SD binding site with similar Cache-domain structures. (a) *Psa* PscD-SD with propionate (b) *Anaeromyxobacter dehalogenans* 4K08 with acetate (c) *Vibrio parahaemolyticus* 4EXO and (d) *Helicobacter pylori* TlpB. Amino acid side chains are shown in sticks representation with carbon atoms in dark grey; ligands are is shown with carbon atoms in light grey. Oxygen and nitrogen atoms are colored red and blue, respectively. Hydrogen bonds are represented by dashed lines (yellow) and the bond distances in Ångstroms are marked on the figure. Hydrophobic interactions are represented as red arcs with spikes. The figure was drawn using LigPlot+. [1].

Reference

1. Laskowski R. A. & Swindells M. B. LigPlot+: multiple ligand-protein interaction diagrams for drug discovery. J. Chem. Inf. Model. (2011) 51, 2778-2786.

Supplementary Figure 7

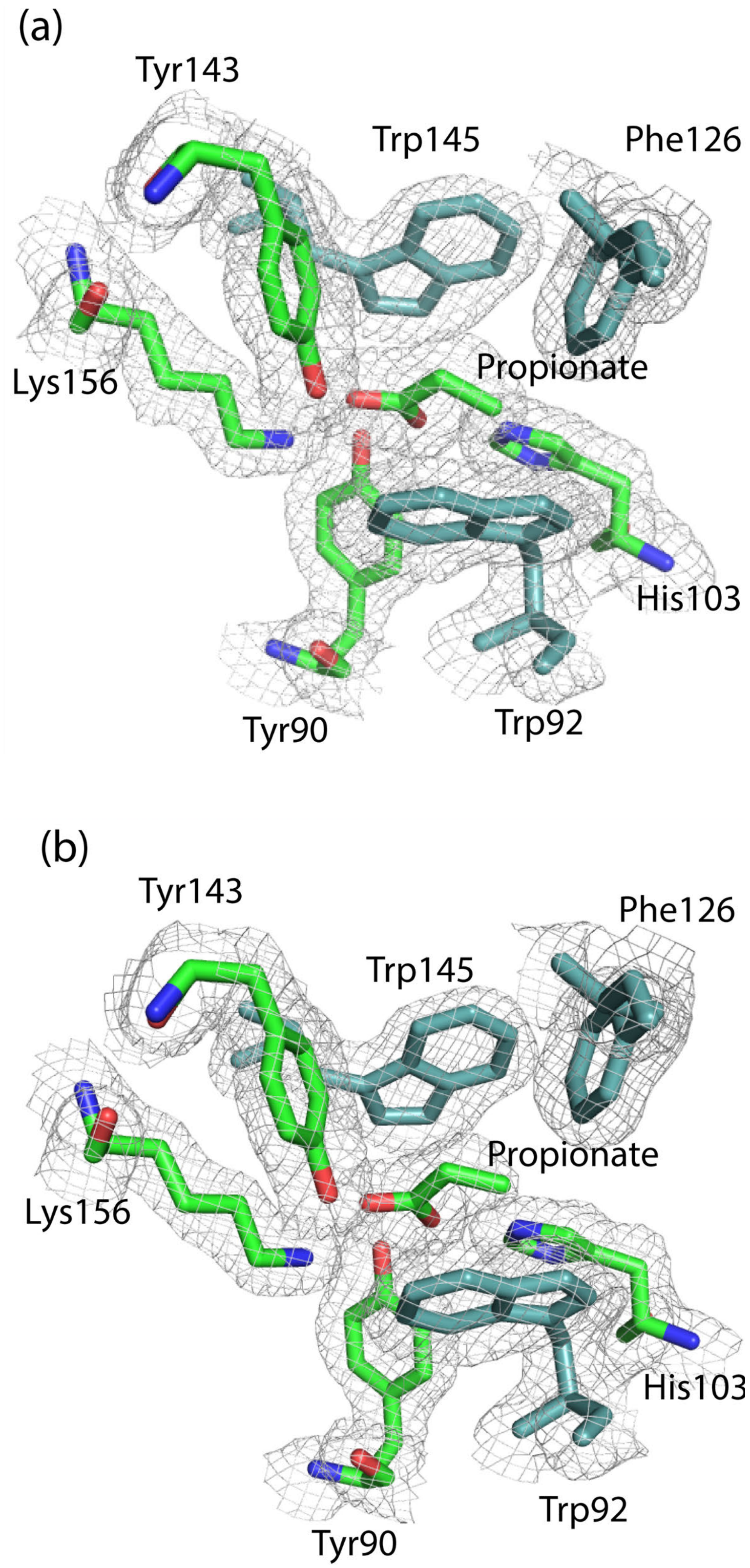

Figure S7. Omit electron density maps of the ligand binding sites. (a) 5g4y 'native' structure and (b) 5g4z propionate soaked structure. A full omit map was generated in the program Phenix by removing 5% of the model iteratively over the whole of the model. Electron density is shown at the level of 1σ. The ligand propionate and the hydrogen bonding residues are colored by chain; green = carbon, blue = nitrogen and red = oxygen. Non-bonding residues are shown in teal.

Supplementary Figure 8

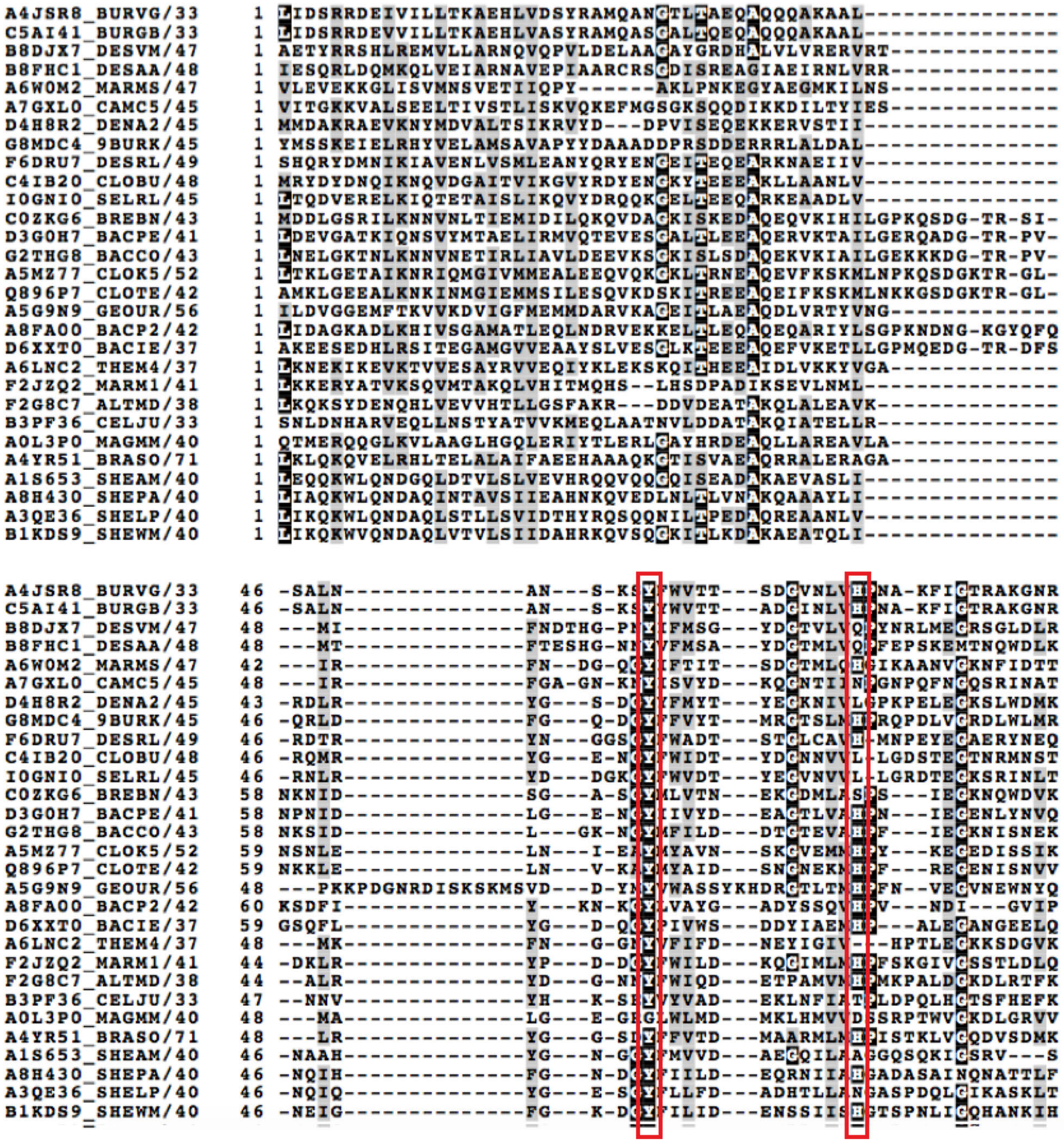

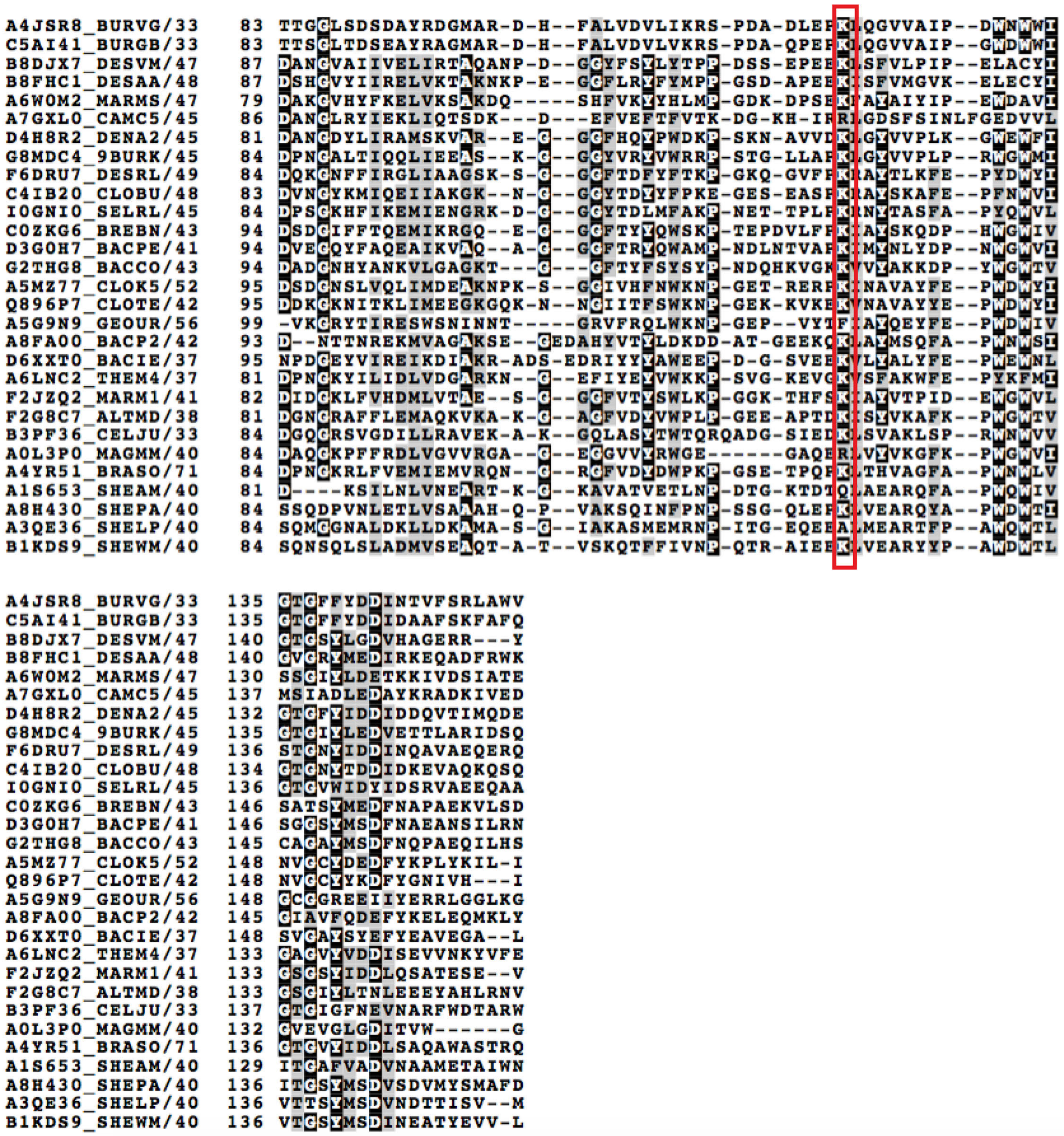

Figure S8. Seed alignment for the single Cache domain family. The Pfam seed alignment for the single Cache domain family (Pfam: sCache_2), containing 29 sequences, was downloaded from pfam.xfam.org. The alignment was shaded using BOXSHADE v 3.21, with a threshold of 0.5 for the fraction of sequences that must agree for shading. The red boxes indicate the conserved residues that are likely involved in carboxylate binding, equivalent to Tyr90, His103 and Lys156 in PscD-SD.

Table S1. The potential ligands tested from Biolog PM plate 1 and the corresponding *Psa* PscD-SD thermal shift assay results.

Well	Compound	Δ Tm
A1	Water	0.00 ± 0.0
A2	L-Arabinose	0.05 ± 0.07
A3	*N*-Acetyl-D-Glucosamine	0.10 ± 0.05
A4	D-Saccaric Acid	0.12 ± 0.05
A5	Succinic Acid	0.10 ± 0.03
A6	D-Galactose	0.17 ± 0.02
A7	L-Aspartic Acid	0.17 ± 0.05
A8	L-Proline	0.07 ± 0.04
A9	D-Alanine	0.12 ± 0.03
A10	D-Trehalose	0.20 ±0.03
A11	D-Mannose	0.17 ± 0.05
A12	Dulcitol	0.19 ± 0.04
B1	D-Serine	0.00 ± 0.00
B2	D-Sorbitol	0.05 ± 0.07
B3	Glycerol	0.10 ± 0.05
B4	L-Fucose	0.12 ± 0.05
B5	D-Glucuronic Acid	0.10 ± 0.03
B6	D-Gluconic Acid	0.17 ± 0.02
B7	D,L,-α-Glycerol Phosphate	0.17 ±0.05
B8	D-Xylose	0.07 ±0.04
B9	L-Lactic Acid	0.12 ±0.03
B10	Formic Acid	0.20 ±0.03
B11	D-Mannitol	0.17 ±0.05
B12	L-glutamic acid	0.15 ±0.05
C1	L-Asparagine	0.00 ±0.00
C2	D-Aspartic Acid	-0.13 ±0.05
C3	D,L-Malic Acid	0.10 ±0.05
C4	D-Ribose	0.12 ±0.05
C5	Tween 20	—
C6	L-Rhamnose	0.1 ± 0.02
C7	D-Fructose	0.17 ± 0.05
C8	Acetic Acid	3.27 ± 0.05
C9	α-D-Glucose	0.12 ± 0.03
C10	Maltose	0.20 ± 0.03
C11	D-Melbiose	0.17 ± 0.05
C12	Thymidine	0.15 ± 0.05
D1	L-Asparagine	0.00 ± 0.00
D2	D-Aspartic Acid	0.05 ± 0.07
D3	D-Glucosaminic Acid	0.10 ± 0.05
D4	1,2-Propanediol	0.12 ± 0.05
D5	Tween 40	—
D6	α-Keto-Glutaric Acid	0.17 ± 0.02
D7	α-Keto-Butyric Acid	0.17 ± 0.05
D8	α-Methyl-D-Galactoside	0.07 ± 0.04
D9	α-D-Lactose	0.12 ± 0.03
D10	Lactulose	0.20 ± 0.03
D11	Sucrose	0.17 ± 0.05
D12	Uridine	0.15 ± 0.05

Well	Compound	Δ Tm
E1	L-Glutamine	0.00 ± 0.00
E2	*m*-Tartaric Acid	0.05 ± 0.07
E3	D-Glucose-1-Phosphate	0.10 ± 0.05
E4	D-Fructose-6-Phosphate	0.12 ± 0.05
E5	Tween 80	—
E6	α-Hydroxy-Glutaric Acid-γ-Lactone	0.17 ± 0.02
E7	α-Hydroxy Butyric Acid	0.17 ± 0.05
E8	β-Methyl-D-Glucoside	0.07 ± 0.04
E9	Adonitol	0.12 ± 0.03
E10	Maltoriose	0.20 ± 0.03
E11	2-Deoxy-Adenosine	0.17 ± 0.05
E12	Adenosine	0.15 ± 0.05
F1	Glycyl-L-Aspartic Acid	0.00 ± 0.00
F2	Cirtric Acid	0.05 ± 0.07
F3	m-Inositol	0.10 ± 0.05
F4	D-Threonine	0.12 ±. 0.05
F5	Fumaric Acid	0.10 ± 0. 03
F6	Bromo Succinic Acid	0.05 ± 0.10
F7	Propionic Acid	2.66 ± 0.11
F8	Mucic Acid	0.07 ± 0.04
F9	Glycolic Acid	1.13 ± 0.00
F10	Glyoxylic Acid	0.86 ± 0.09
F11	D-Cellobiose	0.17 ± 0.05
F12	Inosine	0.15 ± 0.05
G1	Glycyl-L-Glutamic Acid	0.00 ± 0.00
G2	Tricarballylic Acid	-0.03 ± 0.05
G3	L-Serine	0.10 ± 0.05
G4	L-Threonine	0.12 ± 0.05
G5	L-Alanine	0.10 ± 0.03
G6	L-Alanyl-Glycine	0.07 ± 0.08
G7	Acetoacetic Acid	0.63 ± 0.05
G8	*N*-Acetyl-β-D-Mannosamine	0.07 ± 0.04
G9	Mono Methyl Succinate	0.12 ± 0.03
G10	Methyl Pyruvate	0.48 ± 0.13
G11	D-Malic Acid	0.17 ± 0.05
G12	L-Malic Acid	0.15 ± 0.05
H1	Glycyl-L-Proline	0.00 ± 0.00
H2	*p*-Hydroxy Phenyl Acetic Acid	0.15 ± 0.04
H3	*m*-Hydroxy Phenyl Acetic Acid	0.10 ± 0.05
H4	Tyramine	0.12 ± 0.03
H5	D-Psicose	0.10 ± 0.09
H6	L-Lyxose	0.28 ± 0.05
H7	Glucuronamide	0.17 ± 0.06
H8	Pyruvic Acid	1.01 ± 0.03
H9	L-Galactonic Acid-γ-Lactone	0.12 ± 0.03
H10	D-Galacturonic Acid	0.20 ± 0.03
H11	Phenylethyl-amine	0.17 ± 0.05
H12	2-Aminoethanol	0.15 ± 0.05

Data are means and standard errors from three independent experiments. Compounds are listed as the acid form, though many will be present in the deprotonated (conjugate base) form under the assay conditions (pH ~7.0).

— indicates the protein was already unfolded at the initial starting temperature and therefore a Tm could not be calculated.

Table S2. The potential ligands tested from Biolog PM plate 2 and the corresponding *Psa* PscD-SD thermal shift assay results.

Well	Compound	Δ Tm
A1	Water	0.00 ± 0.0
A2	Chondroitin Sulfate C	0.08 ± 0.0
A3	α-Cyclodextrin	0.08 ± 0.0
A4	β-Cyclodextrin	0.45 ± 0.0
A5	γ-Cyclodextrin	0.28 ± 0.07
A6	Dextrin	0.00 ± 0.04
A7	Gelatin	0.03 ± 0.03
A8	Glycogen	0.03 ± 0.05
A9	Inulin	0.08 ± 0.00
A10	Laminarin	0.05 ± 0.03
A11	Mannan	0.10 ± 0.11
A12	Pectin	-0.12 ± 0.06
B1	*N*-Acetyl-D-Galactosamine	0.00 ± 0.00
B2	*N*-Acetyl-Neuraminic Acid	0.08 ± 0.00
B3	β-D-Allose	0.08 ± 0.00
B4	Amygdalin	0.03 ± 0.03
B5	D-Arabinose	0.05 ± 0.05
B6	D-Arabitol	0.00 ± 0.05
B7	L-Arabitol	0.03 ± 0.03
B8	Arbutin	0.03 ± 0.05
B9	2-Deoxy-D-Ribose	0.66 ± 0.05
B10	i-Erthritol	0.05 ± 0.03
B11	D-Fucose	0.10 ± 0.11
B12	2-0- β-D-Galacto-pyranosyl-D-Arabinose	0.10 ± 0.07
C1	Gentiobiose	0.00 ± 0.00
C2	L-Glucose	0.08 ± 0.00
C3	Lactitol	0.08 ± 0.00
C4	D-Melezitose	0.03 ± 0.03
C5	Maltitol	0.05 ± 0.05
C6	α-Methyl-D-Gluoside	0.00 ± 0.04
C7	β-Methyl-D-Glactoside	0.03 ± 0.03
C8	3-Methyl Glucose	0.03 ± 0.05
C9	β-Methyl-D-Glucuronic Acid	0.08 ± 0.00
C10	α-Methyl-D-Mannoside	0.05 ± 0.03
C11	β-Methyl-D-Xyloside	0.10 ± 0.11
C12	Palatinose	0.25 ± 0.21
D1	D-Raffinose	0.00 ± 0.00
D2	Salicin	0.08 ± 0.00
D3	Sedoheptulosan	0.08 ± 0.00
D4	L-Sorbose	0.03 ± 0.03
D5	Stachyose	0.05 ± 0.05
D6	D-Tagatose	0.00 ± 0.04
D7	Turanose	0.03 ± 0.03
D8	Xyitol	0.03 ± 0.05
D9	*N*-Acetyl-D-Glucosaminitol	0.08 ± 0.00
D10	γ-amino butyric acid	0.05 ± 0.03
D11	amino valeric acid	0.26 ± 0.26
D12	Butyric Acid	0.96 ± 0.13
E1	Capric Acid (decanoic acid)	2.24 ± 0.05
E2	Caproic Acid	1.21 ± 0.16
E3	Citraconic Acid	0.08 ± 0.00
E4	Citramalic Acid	0.03 ± 0.03
E5	D-Glucosamine	-0.25 ± 0.03
E6	2-Hydroxy Benzoic Acid	0.00 ± 0.04
E7	4-Hydroxy Benzoic Acid	0.03 ± 0.03
E8	β-Hydroxy Butyric Acid	0.03 ± 0.05
E9	γ-Hydroxy Butyric Acid	0.08 ± 0.00
E10	α-Keto-Valeric Acid	0.23 ± 0.12
E11	Itaconic Acid	-0.05 ± 0.33
E12	5-keto-D-Gluconic Acid	0.10 ± 0.02
F1	D-Lactic Acid Methyl Ester	0.00 ± 0.00
F2	Malonic Acid	0.08 ± 0.00
F3	Melibionic Acid	0.08 ± 0.00
F4	Oxalic Acid	0.03 ± 0.03
F5	Oxalomalic Acid	0.05 ± 0.05
F6	Quinic Acid	0.00 ± 0.04
F7	D-Ribono-1-4-Lactone	-0.20 ± 0.14
F8	Sebacic Acid	0.48 ± 0.09
F9	Sorbic Acid	0.65 ± 0.09
F10	Succinamic Acid	0.18 ± 0.10
F11	D-Tartaric Acid	0.10 ± 0.11
F12	L-Tartaric Acid	0.33 ± 0.29
G1	Acetamide	0.00 ± 0.00
G2	L-Alaninamide	0.08 ± 0.00
G3	*N*-Acetyl-L-Glutamic Acid	0.08 ± 0.00
G4	L-Arginine	0.03 ± 0.03
G5	Glycine	0.05 ± 0.05
G6	L-Histidine	0.00 ± 0.04
G7	L-Homoserine	0.03 ± 0.03
G8	Hydroxy-L-Proline	0.03 ± 0.05
G9	L-Isoleucine	0.08 ± 0.00
G10	L-Leucine	0.05 ± 0.03
G11	L-Lysine	0.10 ± 0.11
G12	L-Methionine	0.43 ± 0.39
H1	L-Ornithine	0.00 ± 0.00
H2	L-Phenylalanine	0.08 ± 0.00
H3	L-Pyroglutamic Acid	0.08 ± 0.00
H4	L-Valine	0.03 ± 0.03
H5	D,L-Carnitine	-0.02 ± 0.02
H6	Sec-Butylamine	0.00 ± 0.04
H7	D,L-Octopamine	0.03 ± 0.03
H8	Putrescine	0.03 ± 0.05
H9	Dihydroxyacetone	0.35 ± 0.14
H10	2,3-Butanediol	0.05 ± 0.03
H11	2,3-Butanone	0.78 ± 0.23
H12	2-Hydroxy-2-Butanone	0.30 ± 0.26

Data are means and standard deviations from three independent experiments. Compounds are listed as the acid form, though many will be present in the deprotonated (conjugate base) form under the assay conditions (pH ~7.0).
